# Isolation and Time Lapse Microscopy of Highly Pure Hepatic Stellate Cells

**DOI:** 10.1155/2015/417023

**Published:** 2015-07-16

**Authors:** Matthias Bartneck, Klaudia Theresa Warzecha, Carmen Gabriele Tag, Sibille Sauer-Lehnen, Felix Heymann, Christian Trautwein, Ralf Weiskirchen, Frank Tacke

**Affiliations:** ^1^Department of Medicine III, RWTH University Hospital Aachen, Pauwelsstrasse 30, 52074 Aachen, Germany; ^2^Institute of Molecular Pathobiochemistry, Experimental Gene Therapy and Clinical Chemistry, RWTH University Hospital Aachen, Pauwelsstrasse 30, 52074 Aachen, Germany

## Abstract

Hepatic stellate cells (HSC) are the main effector cells for liver fibrosis. We aimed at optimizing HSC isolation by an additional step of fluorescence-activated cell sorting (FACS) via a UV laser. HSC were isolated from livers of healthy mice and animals subjected to experimental fibrosis. HSC isolation by iohexol- (Nycodenz) based density centrifugation was compared to a method with subsequent FACS-based sorting. We assessed cellular purity, viability, morphology, and functional properties like proliferation, migration, activation marker, and collagen expression. FACS-augmented isolation resulted in a significantly increased purity of stellate cells (>99%) compared to iohexol-based density centrifugation (60–95%), primarily by excluding doublets of HSC and Kupffer cells (KC). Importantly, this method is also applicable to young animals and mice with liver fibrosis. Viability, migratory properties, and HSC transdifferentiation *in vitro* were preserved upon FACS-based isolation, as assessed using time lapse microscopy. During maturation of HSC in culture, we did not observe HSC cell division using time lapse microscopy. Strikingly, FACS-isolated, differentiated HSC showed very limited molecular and functional responses to LPS stimulation. In conclusion, isolating HSC from mouse liver by additional FACS significantly increases cell purity by removing contaminations from other cell populations especially KC, without affecting HSC viability, migration, or differentiation.

## 1. Introduction

Hepatic stellate cells (HSC) are the main effector cells in liver fibrosis [1]. In homeostatic conditions, they reside in the perisinusoidal space of Dissé, store vitamin A, and are involved in maintaining tissue integrity [2]. In case of liver injury, HSC can be activated by different stimuli such as macrophages [3] or danger-associated signals [4]. Activated HSC were found to release proinflammatory mediators and transdifferentiate into myofibroblasts, which are highly proliferative and produce large amounts of extracellular matrix proteins such as collagen types I and III. This process leads to the excess production of hepatic connective tissue, ultimately leading to hepatic fibrosis, and reduced in liver functionality [5].

Activated HSC are considered one of the major target cells for antifibrotic therapies, because they are the main contributors of hepatic extracellular matrix [6]. In order to study HSC biology and to evaluate therapeutic strategies affecting HSC activation or functionality, primary HSC isolation from human, mouse, or rat liver is an evitable tool in experimental fibrosis research. Early attempts to isolate HSC from mouse or rat livers were based on centrifugal fractionation and/or centrifugal elutriation [7, 8]. Subsequent methods incorporated the simultaneous isolation of different hepatic cell populations based on density gradient centrifugation with Stractan [9]. With the rise of flow cytometry and flow cytometric cell sorting, early attempts for flow cytometric cell sorting were based on the strong sideward scattering of HSC due to the specific intracellular (retinol) droplets [10]. Later strategies incorporated multiplex staining of surface markers and cell sorting to exclude cell types other than HSC from cell purifications. However, the purity of all these strategies for HSC isolation remained disputed, since antibody staining may affect cell populations [11]. Moreover, there is no reliable surface marker known that is generally expressed on HSC and myofibroblasts, which hampers positive selection strategies based on antibody staining [5]. Some surface markers that had been suggested for HSC isolation include platelet-derived growth factor *β* (PDGFR-*β*) or low-affinity nerve growth factor receptor (p75), while other investigators tested intracellular glial fibrillary acidic protein (GFAP) staining to identify Vitamin A^+^ HSC [1, 5, 12]. FACS sorting of HSC has been lately employed by several groups, including ours, by using a UV laser that specifically excites the stored retinol droplets in resting HSC [12–18]. However, it remained unclear whether this technique would alter functional properties of HSC, such as migratory properties relevant to wound healing responses.

The current “gold standard” for HSC isolation is still based on density centrifugation using iohexol (also known under their brand names Nycodenz, Exypaque, or Omnipaque), which separates the HSC due to its physical properties from other hepatic cells and usually results in a high number of viable HSC applicable to cell culture experiments. We hypothesized that, via the “conventional” density gradient method, cell aggregates between HSC and other cell types, especially Kupffer cells (KC), may occur, which in turn result in cellular impurities that could lead to contradictory results on distinct HSC functions. For example, it is heavily debated in the field whether HSC can act as antigen-presenting cells, as studies have led to conflicting results on this topic [11, 19]. Also the notion that HSC strongly respond to bacterial products like lipopolysaccharides (LPS) [20, 21] requires that there is no relevant contamination with macrophages, which are equipped with manifold danger-recognition receptors [22].

In this study, we optimized the “conventional” methodology for the isolation of HSC, based on iohexol density centrifugation, and compared it to an additional step of fluorescence-activated cell sorting (FACS), based on antibodies and UV-autofluorescence, with respect to cellular purity, viability, yield, and cellular characteristics. We further analyzed the behavior of highly pure HSC* in vitro* by studying their cellular morphology and maturation over five days of culture using time lapse microscopy as well as migratory properties in an assay for cell migration and after stimulation with LPS. By implementing an additional step of cell sorting to the current “gold standard” HSC isolation method, our protocol results in significantly improved cellular purity, which helps to clarify HSC functions.

## 2. Materials and Methods

### 2.1. Ethics Statement

All* in vivo* experiments were performed following approval by the State Animal Protection Board at the Bezirksregierung Cologne, Germany. The investigation conforms to the Guide for the Care and Use of Laboratory Animals published by the US National Institutes of Health (NIH Publication Number 85-23, revised 1996).

### 2.2. Mice

C57BL/6J wild-type mice at 40–50 weeks of age, if not stated otherwise, were housed in a specific pathogen-free environment. To induce liver fibrosis, carbon tetrachloride (CCl_4_, 0.6 mL/kg, Sigma-Aldrich, Taufkirchen, Germany) was injected intraperitoneally two times per week for six weeks; control animals received the vehicle (corn oil) [13]. All animal experiments have been approved by the Institutional Review Board and by the German legal authorities (LANUV, Recklinghausen, Germany).

### 2.3. Liver Perfusion, Enzymatic Digestion, and Density Gradient Centrifugation

Mice were anaesthetized using 7 mg/kg body weight xylazine and 105 mg/kg body weight of ketamine. The liver was perfused via the* Vena portae *using a 26 G needle (BD, Franklin Lakes, USA) that was fixed using a Bulldog clamp (Aesculap, Tuttlingen, Germany). Buffers were prewarmed to 37°C and pumped into the liver via the abdominal* Vena portae *and drained via the* Vena cava inferior* using a peristaltic pump at a flow rate of 6.5 mL/minute.

Initially, perfusion buffer 1 (8 g/L NaCl, 400 mg/L KCl, 78 mg/L NaH_2_PO_4_  ×  H_2_O, 151 mg/L NaHPO_4_  ×  2 H_2_O, 2380 mg/L HEPES, 350 mg/L NaHCO_3_, 190 mg/L EGTA, 900 mg/L glucose, and 6 mg/L phenol red, adjusted to pH 7.3–7.4 using 10 N NaOH, sterile filtered, and kept at 4°C until use) was injected into the liver for 4.5 minutes. Second, perfusion buffer 2 (8 g/L NaCl, 400 mg/L KCl, 78 mg/L NaH_2_PO_4_  ×  H_2_O, 151 mg/L NaHPO_4_  ×  2 H_2_O, 2380 mg/L HEPES, 350 mg/L NaHCO_3_, 560 mg/L CaCl_2_  ×  2 H_2_O, and 6 mg/L phenol red, adjusted to pH 7.3–7.4 using 10 N NaOH, sterile filtered, and kept at 4°C until use) was applied for 4.5 minutes and supplemented with 0.5 mg/mL pronase E (Merck, Darmstadt, Germany). Third, perfusion buffer 2 which was supplemented with 0.75 U/mL collagenase P (Roche, Basel, Switzerland) was administered for 4.5 minutes. The perfused liver was removed, and the gall bladder and connective tissue sticking to the liver were detached. HSC were removed by tearing the liver into bits using two tweezers.

The liver cell suspension was further digested for 20 minutes in a 37°C water bath in perfusion buffer 2 supplemented with 0.4 mg/mL pronase E, 1.5 U/mL collagenase P, and 0.02 mg/mL DNase I (Roche, Basel, Switzerland), adjusted to a pH of 7.2–7.4 using NaOH. Cells were filtered using a 70 *μ*m nylon gaze and rinsed using perfusion buffer 2 tempered at 4°C and centrifuged for ten minutes at 4°C at 600 g. The pellet was then washed using 4°C Gey's buffered salt solution (GBSS) (exact composition: 370 mg/L KCl, 210 mg/L MgCl_2_  ×  6 H_2_O, 70 mg/L MgSO_4_  ×  7 H_2_O, 75 mg/L Na_2_HPO_4_  ×  2 H_2_O, 30 mg/L KH_2_PO_4_, 991 mg/L glucose, 227 mg/L NaHCO_3_, 225 mg/L CaCl_2_  ×  H_2_O, 8000 mg/L NaCl, and 6 mg/L phenol red, adjusted to pH 7.3–7.4, sterile filtered, and kept at 4°C until use) supplemented with 0.01 mg/mL DNase I. Cells were then centrifuged for ten minutes at 4°C at 600 g and suspended in the same buffer (without NaCl, otherwise identical) that was supplemented with iohexol (Nycodenz, Axis-Shield, Dundee, Scotland) to a final concentration of 8%.

The mixture was then pipetted to the ground of a falcon containing GBSS and centrifuged at 4°C and 1500 g for 22 minutes without brake. The interphase containing enriched HSC between the GBSS and iohexol layer was removed and washed using 4°C tempered GBSS (for cell culture) or 4°C Hank's complete (Hank's buffered salt solution without calcium or magnesium containing 10 mM 4-(2-hydroxyethyl)-1-piperazineethanesulfonic acid (HEPES), 0.06% bovine serum albumin (BSA), and 0.3 mM EDTA, adjusted to pH 7.3–7.4 (for FACS). The cells were then washed for ten minutes at 600 g and the supernatant was removed.

In case of optional F4/80 staining (here only done to identify HSC-KC doublets) that is not required routinely, the cell pellet was resuspended in 4°C FACS staining buffer (one part of phosphate-buffered saline without calcium or magnesium and one part of Hank's complete, supplemented with each 1% of mouse, human, and rabbit serum, and 1% BSA). Staining with anti-F4/80 antibody (diluted 1 : 400) conjugated with PE-Cy7 rat anti-mouse antibody was done for 30 minutes; antibody and staining buffer were removed by one time of washing in Hank's complete for ten minutes at 4°C and 500 g without brake and suspending in the same solution.

### 2.4. Fluorescence-Activated Cell Sorting

Cell sorting was done using a BD FACS Aria II SORP Cell Sorter (BD Biosciences, Franklin Lakes, NJ, USA). The pellet was resolved in 4°C Hank's complete and filtered using 40 *μ*m nylon gaze. The sorting of the HSC required excitation via UV laser and measuring the emission in the Indo-1 channel based on a 505 nm long pass and a 530 ± 30 nm band-pass filter. The sample loading port was set to 4°C and 300 rpm. We used a 100 *μ*m nozzle and a pressure of 20 psi. HBSS with calcium or magnesium was used as sheath fluid. The flow rate was set to 5000 events per second and the threshold was adjusted to 5000. The precision mode purity was set up using a yield mask of 32, a purity mask of 32, and a phase mask of 0. The collection device was set to 4°C. The collection tube was a 5 mL glass tube that contained 1 mL of Hank's BSS without calcium or magnesium, 10 mM HEPES, and 20% of fetal bovine serum (FBS). After sorting, cells were centrifuged for 10 minutes at 4°C and at 750 g without brake.

### 2.5. Cell Culture, Viability, and Purity

Cells were counted using a hemocytometer (Neubauer chamber) and 0.4% trypan blue (Sigma-Aldrich, St. Louis, MO, USA). The purity after sorting was determined by a BD FACS Aria II SORP Cell Sorter (BD Biosciences, Franklin Lakes, NJ, USA) and a hemocytometer. The cell pellet was suspended in DMEM cell culture medium supplemented with 25 mM HEPES, 4.5 g/L glucose, 4 mM glutamine, 1% penicillin/streptomycin, and 10% FBS (all Lonza, Basel, Switzerland). Cell culture was done in 24-well plates (Greiner, Kremsmünster, Austria) using 40,000 cells per well in a volume of 1 mL medium.

### 2.6. Fluorescence Microscopy

To stain cells for actin, phallotoxin staining was done. Therefore, cells were grown on glass slides that were precoated with poly-L-lysine as described earlier [23]. Cells were washed before and after each step using 4°C* Hank's buffered salt solution (HBSS)*. Cells were fixed using 4% paraformaldehyde for 20 minutes at 4°C. Permeabilization was done using 0.2% triton X-100 for 4 minutes at 4°C. A solution of 3% of BSA dissolved in PBS was used to inhibit unspecific binding. Actin staining was done for 20 minutes at room temperature (21°C) using rhodamine phalloidin (Invitrogen, Carlsbad, CA, USA) which was diluted 1 : 40 in PBS. Desmin staining was done by the same methods for fixation, permeabilization, and blocking. The primary antibody was antidesmin (Novus Biologicals, Littleton, Colorado, USA). Cells were then blocked again and the secondary antibody was Cy3 donkey anti-rabbit (Jackson Immuno Research, Bethesda, MD, USA) diluted 1 : 100 in 3% BSA in PBS for 90 minutes at room temperature. Nuclear staining was done using 0.1 *μ*g/mL 4′,6-diamidino-2-phenylindole (DAPI) in HBSS for 5 minutes at room temperature. Finally, slides were mounted using Vectashield Mounting medium (Vector laboratories, Burlingame, CA, USA). Micrographs were made using a DMLB (Leica, Microsystems, Vienna, Austria).

### 2.7. Staining of Liver Sections

Paraffin-embedded liver sections were cleared from paraffin using xylene and ethanol in ascending line and distilled water for all staining procedures. Hematoxylin and eosin (H&E) staining was done by 15 minutes of incubation in hematoxylin, rinsing for 10 minutes using warm tap water, and 5 minutes of staining with eosin. Sirius red staining was performed using 100 mg Sirius Red (Polysciences Inc., Warrington, USA) in 100 mL saturated picric acid for staining, with the pH adjusted to 2 using 2 N NaOH. To differentiate, sections were incubated for two minutes in 0.01 N HCl and rinsed with tap water. Morphometric quantification of collagen fibres was done using Image J. Staining for *α* smooth muscle actin (*α*SMA) was done using the antibody M0851 and the Animal Research Kit, according to the instructions of the manufacturer (all Dako, Hamburg, Germany). All sections were dehydrated using a descending order of ethanol and xylene.

### 2.8. Quantitative Gene Expression Analysis

To isolate RNA from cells, peqGOLD (peqLab, Erlangen, Germany) was added to cell pellets, and RNA was purified as described before [24]. Complementary DNA was generated from 100 ng of RNA using the Transcriptor first strand cDNA synthesis kit (Roche, Basel, Switzerland). The quantitative real-time polymerase chain reaction (PCR) was performed using SYBR Green Reagent (Invitrogen, Carlsbad, CA, USA) using a 7500 PCR system (Applied Biosystems, Carlsbad, CA, USA). Exon-spanning primers were used, and *β*-actin regulation of transcripts was employed to normalize gene expression. Primer sequences are available upon request.

### 2.9. Time Lapse Microscopy and Migration Assays

Micrographs were taken using an Axio Observer Z1 equipped with an Axio Cam MR and an XLmulti S1 DARK LS incubator, providing identical conditions like in a normal incubator. To process data, we used the ZEN pro. 2012 software (Carl Zeiss MicroImaging GmbH, Göttingen, Germany). Pictures were made every 15 minutes. To evaluate the effects of cell culture on HSC proliferation, we normalized the cell number data to the starting point.

To study HSC migration into a defined area, we used culture inserts for self-insertion (IBIDI, Martinsried, Germany) that were inserted before adding the cells to 24-well plates (Greiner, Kremsmünster, Austria). Cells grow among these inserts and, to initiate the migration experiment, the inserts were removed and cells began to move to the empty space (similar to a “scratch assay,” but with a well-defined area for the cells to migrate and as a big advantage, no cells are harmed in this assay).

### 2.10. Statistical Analysis

Statistical analysis was performed using Graph Pad Prism 5.0. Unpaired* t*-tests were performed to test significance of data.

## 3. Results

### 3.1. Effect of Additional Sorting on Stellate Cell Purity, Viability, and Differentiation

**Figure 1:**
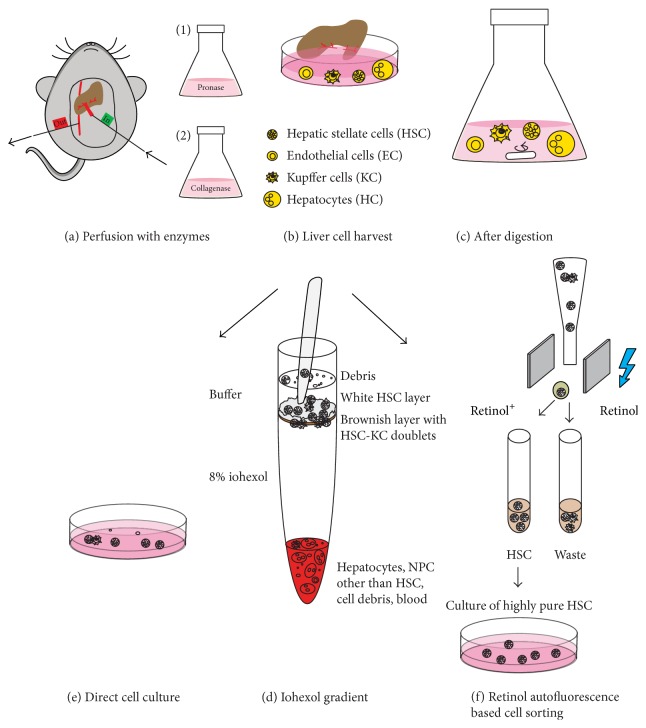
Optimization of the isolation of primary hepatic stellate cells (HSC) based on iohexol density gradient centrifugation and fluorescence-activated cell sorting (FACS) (schematic depiction). In both strategies for cell purification, the mouse is anaesthetized before surgery, and the liver is then perfused via the* Vena portae *and drained through the* Vena cava inferior *using a two-step perfusion of the enzymes pronase and collagenase (a). Liver cells are harvested by gently tearing the liver into bits (b), followed by a postdigestion using a combination of both enzymes (c). The liver cells are subjected to iohexol density gradient centrifugation, after which HSC and Kupffer cells are located in the interphase between iohexol and buffer (d). The enriched HSC layer containing HSC, HSC-Kupffer cell doublets, and cellular debris can be used directly for cell culture of HSC (e) or can be cleared from HSC-Kupffer cell doublets and cellular debris using FACS based on the autofluorescence of retinol, using the UV laser of the cell sorter, resulting in highly pure HSC (f).

We first compared the standard method for HSC isolation to an optimized protocol based on additional sorting of HSC. This standard method includes digesting the liver in anaesthetized mice using pronase and collagenase containing buffers. Pronase reduces the abundance of other liver cells, especially of liver sinusoidal endothelial cells and hepatocytes (Figure 1(a)). Liver cells are then harvested into a cell culture dish (Figure 1(b)), and a postdigestion step is done (Figure 1(c)). The cellular mixture is then subjected to iohexol-based density centrifugation (Figure 1(d)). Cells can be cultured directly (Figure 1(e)), as shown by others [25], or be subjected to an additional step of FACS (Figure 1(f)). The FACS-based isolation of primary HSC from mouse livers has been reported from several groups [12–18] with considerable variations in the exact gating strategy.

**Figure 2:**
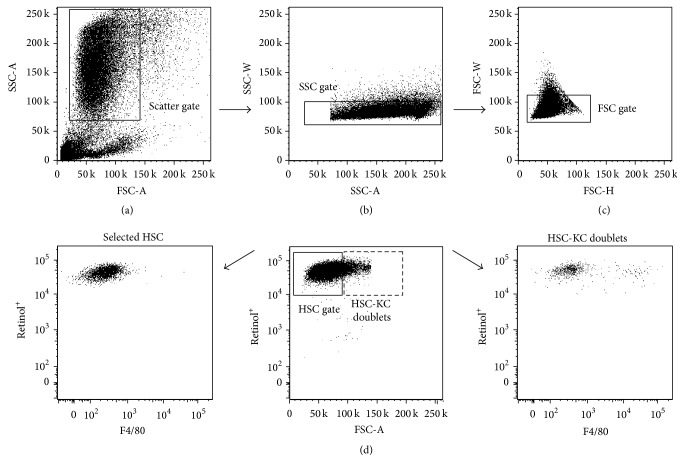
Gating strategy for the purification of hepatic stellate cells using fluorescence-activated cell sorting. Cells are first gated based on their forward and sideward scattering (a), doublets are excluded from sideward (b) and forward scattering (c), and hepatic stellate cells (HSC) are selected based on the UV light excitation of retinol (vitamin A) (d). A detailed cell-type specific staining of the Kupffer cell marker F4/80 demonstrated that the large (here: FSC-A > 100 in the plot, dotted gate) retinol^+^ cells are Kupffer cell- (KC-) HSC doublets that stain positive for F4/80 (right hand side), whereas the smaller retinol^+^ cells are F4/80^−^ HSC (left hand side). By placing a sorting gate as depicted in (d) (middle plot, black line), selected HSC (left plot) are pure and do not contain contaminating KC.

Our FACS gating strategy is based on selecting cells that exhibit a high extent of sideward scattering due to their vitamin A droplets (Figure 2(a)). The next step consists of excluding cell doublets (Figures 2(b) and 2(c)). The most decisive step in our method is the selection of HSC based on their emission of retinol-based autofluorescence via UV excitation (Figure 2(d)). In the protocol, it is further important to exclude the larger retinol^+^ cells (larger cells exhibit a higher forward scattering, FSC) from the HSC gate as these are HSC-KC doublets indicated by their expression of F4/80 (Figure 2(d), right side), whereas the smaller retinol^+^ cells are F4/80^−^ HSC (Figure 2(d), left hand side).

**Figure 3:**
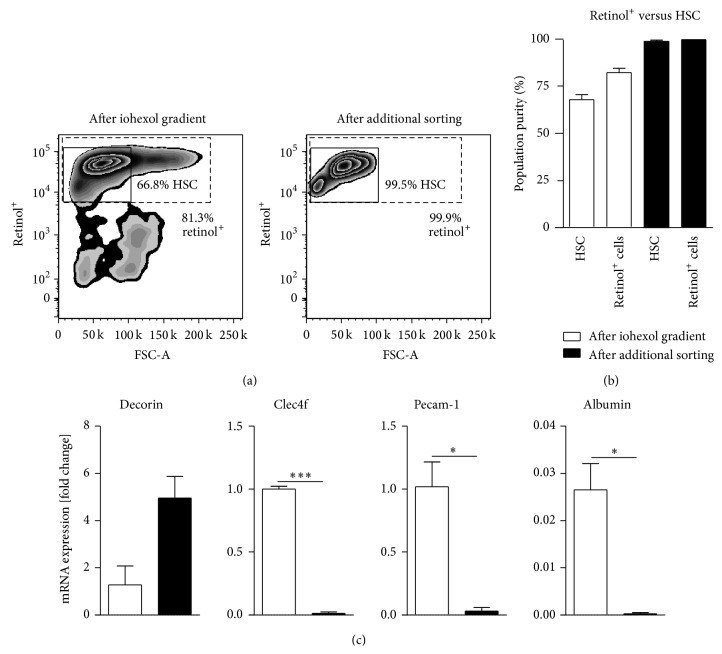
Comparison of the purity of hepatic stellate cells isolated via iohexol gradient without or with subsequent cell sorting. Purity of hepatic stellate cells (HSC) before (left top) and after fluorescence-activated cell sorting (FACS, right top) (a) based on their retinol autofluorescence only (dashed line) or additional exclusion of HSC-Kupffer cell doublets (highly pure real HSC, straight line). The statistical summary of retinol^+^ cells compared to HSC (with HSC-KC doublets excluded) is depicted (b). Analysis of purity using cell type-specific markers for major cell populations. Decorin is considered as a marker for hepatic stellate cells, C-type lectin domain family 4f (Clec4f) is a gene expressed by Kupffer cells, the platelet/endothelial cell adhesion molecule 1 (Pecam-1, CD31) is mainly expressed by endothelial cells, and albumin is a marker for hepatocytes (c). Data are given relative to the expression of *β*-actin of the cells derived from perfusion and digestion. Mean data ± SD of *n* = 4 independent experiments.

Using the iohexol density gradient centrifugation as standard method of HSC purification, we normally observe HSC purities of 60–95% HSC, strongly depending on the batch number of the enzymes (pronase, collagenase) used to digest the liver (which therefore has to be pretested extensively), the age of mice, genetic background, and the gender. By direct comparison, FACS of HSC resulted in final purities of up to 99.5% compared to 66.8% after iohexol-based density centrifugation only (Figure 3(a)). Earlier studies that were claiming to reach 95% or higher levels of purity in conventional HSC isolation may have reported on retinol^+^ cells, which however might contain a considerable quantity of HSC-KC doublets [25]. As shown in Figure 2(d), these retinol^+^ HSC-KC doublets cannot be removed completely by density gradient but only by stringent gating in flow cytometric cell sorting (Figure 2(d)).

This became more evident by statistical evaluations of four independent experiments, which showed that the number of retinol^+^ cells was much higher than the number of pure HSC singlets (Figure 3(b)). Furthermore, the HSC derived from sorting were highly viable indicated by 89.3% living cells, similar to that after the iohexol gradient as retrieved from trypan blue staining (90%) (detailed data not shown). To our experience, it is not reliably possible to discriminate between singular HSC and HSC-KC doublets by microscopic analysis but it requires FACS to remove doublets (Figure 2(d)).

The dramatic improvement in HSC purity was corroborated by quantitative real-time PCR analyses. After flow cytometry-based sorting, mRNA expression of the HSC-specific decorin was further increased (Figure 3(c)) compared to standard density centrifugation isolation. On the other hand, the expression of mRNA characteristic of other hepatic cell types, such as the C-type lectin domain family 4f (Clec4f), which is expressed by macrophages, the platelet endothelial cell adhesion molecule 1 (Pecam-1, CD31) exhibited by liver sinusoidal endothelial cells, and albumin, reflective of hepatocytes, were strongly and significantly reduced by additional FACS (Figure 3(c)).

**Figure 4:**
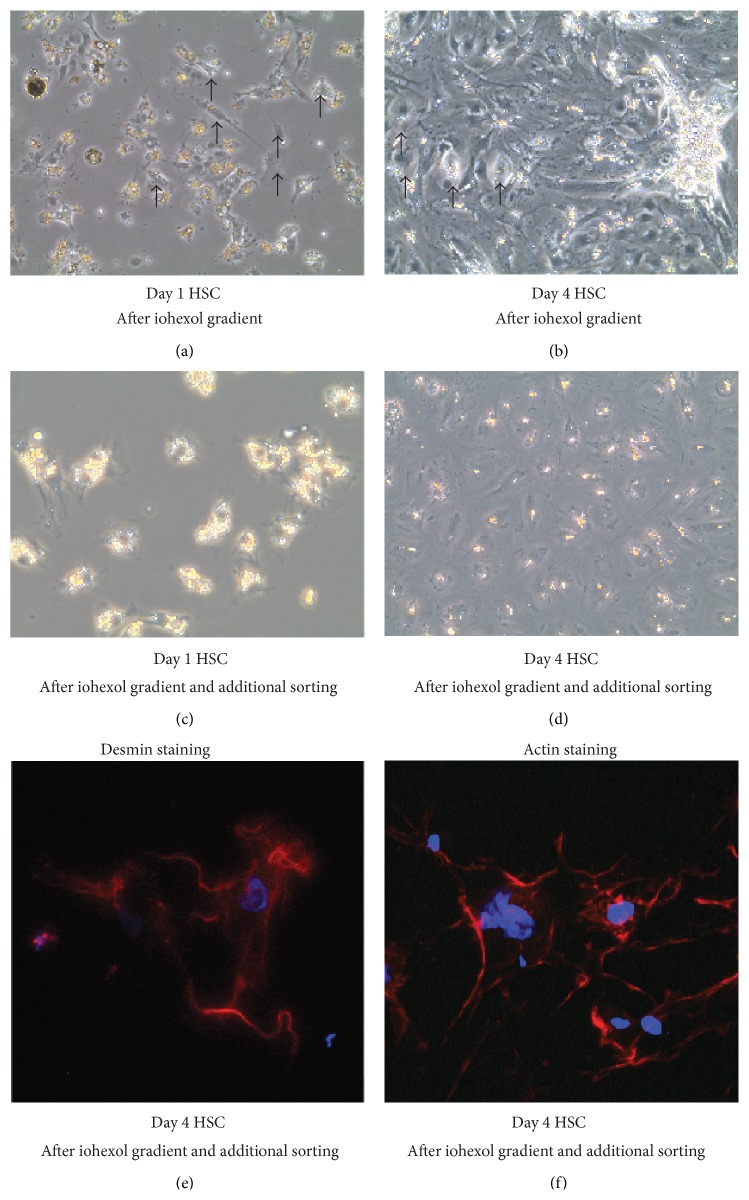
Cultures of hepatic stellate cells isolated via iohexol density gradient centrifugation without or with subsequent cell sorting. Hepatic stellate cells (HSC) were isolated from 40-week-old C57BL/6J mice using enzymatic digestion of the liver based on pronase and collagenase, followed by density gradient centrifugation in 8% iohexol. Cells were cultured directly after the gradient for one day (a) and four days (b), where Kupffer cells (indicated by arrows) can be found in the HSC culture. Highly pure HSC after additional fluorescence-activated cell sorting (FACS) after one (c) and four days of culture are shown (d). Expression analysis of desmin (e) and phalloidin (f) of HSC after four days of culture, indicating proper maturation of HSC.

To further investigate the functionality of HSC after additional cell sorting* in vitro*, we isolated HSC via iohexol gradient and used half of these cells for an additional step of purification with FACS. Both cell isolates were subsequently cultured for up to four days. If only the iohexol gradient was performed, KC were found in the culture dishes after one day of culture (Figure 4(a)), and, more visibly, after four days, HSC could hardly be identified due to excessive growth of diverse cell types other than HSC, with KC being noticeable (Figure 4(b)). Performing an additional step of FACS-based cell isolation resulted in a significantly higher purity of cell populations, and HSC could clearly be identified due to their retinol droplets (Figure 4(c)). After four days of culture, HSC could still be identified and exhibited a rather stretched morphology, whereas KC or other contaminating cell types could not be observed (Figure 4(d)). Immunofluorescent staining for desmin (Figure 4(e)) and actin (Figure 4(f)) showed that the FACS-isolated cells expressed these characteristic markers of activated HSC at day 4, indicating that the sorting procedure did not negatively impact HSC differentiation* in vitro*.

### 3.2. Suitability of the Methodology for Stellate Cell Isolation from Experimental Liver Injury Models

**Figure 5:**
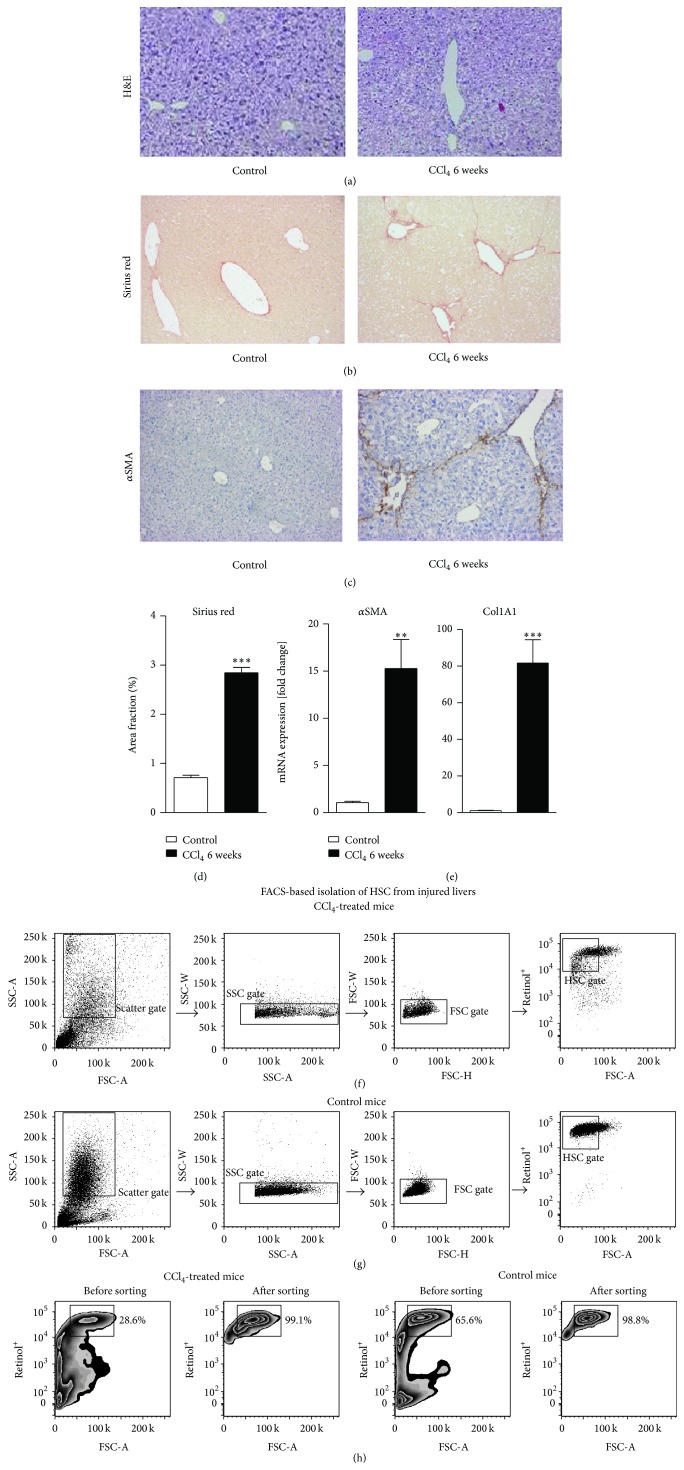
Hepatic stellate cell activation* in vivo* and isolation of hepatic stellate cells from livers of injured mice. Chronic toxic liver injury was induced in 8-week-old C57BL/6 mice by 6 weeks of carbon tetrachloride (CCl_4_) treatment, and control treatment was done using corn oil. Mice were sacrificed 48 hours after the last injection of (CCl_4_) and liver sections were stained for hematoxylin eosin (a), Sirius red (stains collagen fibres, a hallmark of fibrosis) (b), and *α* smooth muscle action (*α*SMA) which targets activated hepatic stellate cells, mediators of fibrosis. Morphometric quantification of Sirius red confirms fibrosis progression (d). Quantitative real-time PCR indicates upregulation of collagen 1 (Col1A1) and *α*SMA mRNA in liver sections (e). Application of the gating strategy of fluorescence-activated cell sorting (FACS) to isolate hepatic stellate cells from livers of mice that underwent six weeks of repetitive CCl_4_-based liver injury (f) compared to vehicle corn oil-treated control mice (g). Flow cytometric analysis of HSC purity before and after sorting demonstrated that the purification was successful (h). Mean ± SD of three independent experiments, *n* = 12 for control and 16 for fibrotic mice; ^*∗∗∗*^ *P* < 0.001, ^*∗∗*^ *P* < 0.005, and ^*∗*^ *P* < 0.05 (unpaired Student's *t*-test).

To outline the activation of HSC* in vivo*, chronic toxic liver injury was induced in 8-week-old C57BL/6 mice using CCl_4_ for six weeks and twice weekly, a standard method for fibrosis induction [13]. Liver damage was associated with necrotic areas and characteristic fibrotic bridging (Figure 5(a)), whereas Sirius red staining which marks collagen reflective of fibrosis confirmed fibrosis progression in the animals treated with CCl_4_ (Figure 5(b)), similar to *α*SMA which stains for activated HSC (Figure 5(c)). Morphometric quantifications of Sirius red revealed a significant increase in the fibrotic areas in the liver of fibrotic mice (Figure 5(d)). Real-time PCR confirmed fibrosis development on the level of collagen 1 and *α*SMA mRNA expression (Figure 5(e)). To investigate whether our method is suitable to isolate HSC from the livers of younger control and diseased mice, HSC were then isolated from these via iohexol density gradient centrifugation and additional FACS. We observed that the gating strategy could also be applied to younger diseased (Figure 5(f)) and control mice (Figure 5(g)), which are known to have fewer numbers of HSC and in which HSC might contain fewer retinol droplets. In case of the diseased mice, the sorting step appeared to be even more important than in case of healthy mice, because after CCl_4_ treatment the amount of debris or other cells compared to HSC (28.6%) was much higher than that for control littermates that were treated with the vehicle corn oil (65.6%) (Figure 5(h)). FACS helped to get rid of cellular debris relating to the toxic effects of CCl_4_ and made it possible to increase the purity up to 99.1% (Figure 5(h)), thereby allowing comparable purity to that of healthy mice.

### 3.3. Functional Studies of FACS-Isolated Stellate Cells* In Vitro*

**Figure 6:**
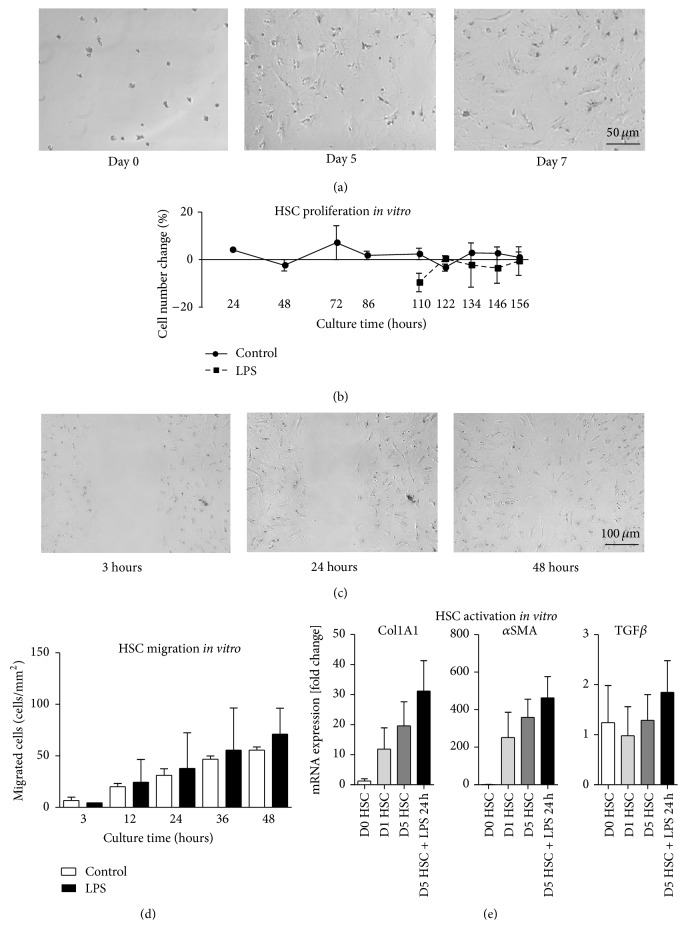
Hepatic stellate cell functionality* in vitro*. Hepatic stellate cells (HSC) were isolated from 40-week-old C57BL/6J mice using enzymatic digestion of the liver based on pronase and collagenase, followed by density gradient centrifugation in 8% iohexol and fluorescence-activated cell sorting (FACS). The HSC were cultured in DMEM with 10% fetal calf serum, and some plates were stimulated with lipopolysaccharides (LPS) at 100 ng/mL (after five days of culture) for another 48 hours. Changes in the cell number during culture were determined from time lapse microscopy (a) and statistical summary (b). HSC were cultured for five days on tissue culture-treated polystyrene in DMEM with 10% fetal calf serum including culture inserts for self-insertion (“scratch assay”). To start horizontal migration, the plastic inserts were removed and HSC migrated (c) and were quantified using software (d). HSC were cultured for designated periods and quantitative real-time PCR was performed to study the expression of *α* smooth muscle actin (*α*SMA), collagen 1 (Col1A1), or the transforming growth factor *β* (TGF*β*) as markers of HSC activation. Gene expression was normalized to *β*-actin expression of cells that were lysed directly after isolation at day zero (e). Mean ± SD of three independent experiments.

To further characterize the HSC* in vitro*, we performed time lapse microscopy under different experimental conditions. During the first five days of HSC culture, retinol droplets moved within the HSC, but we did not observe proliferation of HSC (Video 1 in Supplementary Material available online at http://dx.doi.org/10.1155/2015/417023 and Figures 6(a) and 6(b)). During days five to seven, motility of intracellular retinol droplets was increased, but no HSC proliferation was noted (Video 2 and Figures 6(a) and 6(b)).

It is well established that HSC respond to bacterial products like lipopolysaccharide (LPS) via toll-like receptor 4 signaling [4]. When LPS was added to the cultures at the fourth day of culture for 24 hours, no changes in HSC morphology or in the spontaneous migration of HSC in the culture dishes were observed (Video 3, Figure 6(b)). A longer LPS incubation period from days five to seven did not result in any morphological differences compared to control conditions either (Figure 6(b) and Video 4). To further study HSC functionality, we performed a horizontal migration assay with the HSC (similar to a “scratch assay,” but using culture inserts which results in well-defined regions) at day five until day seven of culture without (Video 5 and Figure 6(c)) and with LPS stimulation (Video 6 and Figure 6(d)). FACS-isolated HSC showed horizontal migration, and the migratory capacity of the HSC was slightly enhanced by LPS (Figure 6(d)).

To further study the differentiation and effector functions of HSC* in vitro*, we isolated mRNA from cells directly after cell sorting, after one day of culture, after five days, and after an additional stimulation with LPS at day four until day five of culture. We found that the HSC-characteristic activation markers collagen 1 (Col1A1) and *α*SMA were upregulated starting at the first day of culture and further increase at day 5, but with comparatively low increase after treatment with lipopolysaccharides (LPS). The transforming growth factor *β* (TGF*β*), however, was only weakly affected during culture (Figure 6(e)).

## 4. Discussion

Mechanistic studies in liver fibrosis research using highly pure HSC rely on a robust method that guarantees cellular purity and functionality. However, especially “conventional” HSC isolation suffers from high levels of variation caused by the batch-dependent quality of the enzymes used for liver digestion, age, gender, genetic background, and the treatment of the mice [5]. The FACS-based sorting of HSC has been reported from several groups, including ours, with considerable variations in the protocols and without comprehensive analyses on the functional properties of FACS-isolated HSC [12–18]. Many researchers in the field believe that FACS-based cell sorting is insufficient to retrieve high enough HSC numbers for functional* in vitro* experiments and, furthermore, may affect the viability of HSC.

In this study, we demonstrate that FACS is actually required to retrieve highly pure and functional HSC and that this methodology allows functional* in vitro* experimentations with sufficient cell numbers and unaffected viability over at least one week of culture. Moreover, this technique is also applicable to the isolation from young animals (e.g., 12 weeks of age) as well as to mice subjected to standard liver injury models like repetitive CCl_4_ administration over 6 weeks. Especially the isolation of HSC from fibrotic livers using the density gradient centrifugation method without FACS has yielded conflicting results, because gene array analyses from these HSC did not match with culture-activated HSC [26]. At this time, the authors found that the addition of KC or LPS to cultured HSC shifted the gene expression pattern towards the* in vivo* activation, suggesting that macrophages and inflammatory cytokines drive HSC activation* in vivo* [26]. However, our data show that, in addition to other nontarget cells, especially doublets of HSC with KC can considerably “contaminate” primary HSC isolates from mouse liver, if no additional FACS-based sorting is performed. Therefore, it is important to exclude that such doublets confounded prior conclusions on HSC gene expression profiles from fibrotic livers.

Along the same line, a solid body of literature exists demonstrating that HSC isolated from mouse or rat livers can produce large amounts of proinflammatory mediators synthesized by HSC [27] and respond vividly to bacterial products like LPS via toll-like receptor recognition [4]. Similarly, LPS was also found to activate human HSC* in vitro* [28]. It will be important to determine whether some of the LPS responsiveness reported for HSC might be partly related to contaminations with KC, which are known to be both efficient producers and responders to inflammatory mediators [22]. Using highly pure HSC cultures after FACS-based isolation, LPS had very limited effects on HSC behavior such as spontaneous migration as well as transdifferentiation or proliferation.

Nevertheless, one should keep in mind that, upon contact with the tissue culture material, HSC begin to mature and do not reflect quiescent HSC but “culture-activated” HSC [29]. This is well reflected by the fact that HSC, isolated either by conventional methodology or with additional FACS, significantly upregulate activation and myofibroblast differentiation markers upon culture.

Another controversial aspect of HSC biology relates to the question whether HSC derived from noninjured liver microenvironment proliferate* in vitro*. It is known that LPS-stimulated peripheral blood mononuclear cells such as monocytes can stimulate HSC proliferation [30]. Moreover, different profibrogenic stimuli, such as TGF-*β*, can stimulate HSC proliferation* in vitro* [31]. However, it was not clear whether highly pure FACS-isolated HSC alone would proliferate. In our hands, highly pure HSC do not proliferate spontaneously, suggesting that they rely on external stimuli such as cytokines from inflamed liver or cell-cell contacts with macrophages [3]. Furthermore, one has to consider that also HSC exhibit heterogeneity and it was reported that retinol^−^ HSC proliferate whereas retinol^+^ cells do not [32].

In conclusion, we developed and validated an optimized isolation procedure for primary HSC from mouse livers, which results in a highly pure, viable, and functionally active population of HSC. Upcoming studies should validate controversial basic studies to unravel to which extent contaminations especially with KC may have confounded earlier conflicting data on HSC biology, as earlier studies suggest that KC release molecules which induce HSC proliferation [33].

## Supplementary Material

Time lapse microscopical studies of murine hepatic stellate cell maturation, their reactions towards the exposure to lipopolysaccharides, and their migrational behavior.
